# Ethnobotanical survey of medicinal and ritual plants utilized by the indigenous communities of Benguet province, Philippines

**DOI:** 10.1186/s41182-024-00624-1

**Published:** 2024-09-10

**Authors:** Janna R. Andalan, Alissa Jane S. Mondejar, Nanette Hope N. Sumaya, Jaime Q. Guihawan, Ma. Reina Suzette B. Madamba, Carlito Baltazar Tabelin, David Guilingen, Florifern C. Paglinawan, Kryzzyl M. Maulas, Isidro Arquisal, Arnel B. Beltran, Aileen H. Orbecido, Michael Angelo Promentilla, Dennis Alonzo, Pamela Flynn Pisda, Alleah Ananayo, Marlon Suelto, Irish Mae Dalona, Vannie Joy Resabal, Robin Armstrong, Anne D. Jungblut, Ana Santos, Pablo Brito-Parada, Yves Plancherel, Richard Herrington, Mylah Villacorte-Tabelin

**Affiliations:** 1 Center for Natural Products and Drug Discovery, PRISM, Mindanao State University–Iligan Institute of Technology, Iligan City, Philippines; 2 Center for Microbial Genomics and Proteomics Innovation, PRISM, Mindanao State University–Iligan Institute of Technology, Iligan City, Philippines; 3 Center for Biodiversity Studies and Conservation, PRISM, Mindanao State University-Iligan Institute of Technology, Iligan City, Philippines; 4 Department of Biological Sciences, College of Science and Mathematics, Mindanao State University–Iligan Institute of Technology, Iligan City, Philippines; 5 Department of Materials and Resources Engineering and Technology, College of Engineering, Mindanao State University–Iligan Institute of Technology, Iligan City, Philippines; 6 Resource Processing and Technology Center, REIT, Mindanao State University–Iligan Institute of Technology, Iligan City, Philippines; 7 Department of Chemical Engineering, De LaSalle University, Manila, Philippines; 8 School of Education, University of New South Wales, Sydney, Australia; 9 University of the Philippines, Los Baños, Laguna, Philippines; 10 College of Arts and Social Sciences, Mindanao State University–Iligan Institute of Technology, Iligan City, Philippines; 11 Department of Science, Natural History Museum, London, UK; 12 Department of Earth Science and Engineering, Imperial College London, London, UK

**Keywords:** Kankanaey, Ibaloi, Kalanguyas, Ilocano, Tublay

## Abstract

**Background:**

The Sto. Niño site in Benguet province, Philippines was once a mining area that has now been transformed into an agricultural land. In this area, there has been significant integration of the three indigenous people (IPs) Ibaloi, Kankanaeys and Kalanguyas with the Ilocano community. These IPs safeguard biodiversity and traditional knowledge, including medicinal plant use. However, the documentation of these plant species and their medicinal applications has not been systematic, with the resultant loss of knowledge across generations. This study aims to document the medicinal and ritual plants used by the indigenous communities at the site, in order to preserve and disseminate traditional medicinal knowledge that would otherwise be lost.

**Methods:**

Ethnobotanical data were collected in Sto. Niño, Brgy. Ambassador, Municipality of Tublay, Benguet, Philippines, and collected through semi-structured interviews, together with focus group discussions (FGD). A total of 100 residents (39 male and 61 female) were interviewed. Among them, 12 were key interviewees, including community elders and farmers, while the rest were selected through the convenience and snowball technique. Demographic information collected from the interviewees included age, gender, and occupation. Ethnobotanical information collected focused on medicinal plants, including the specific parts of plants used, methods of preparation, modes of treatment, and the types of ailments treated. Ethnobotanical quantitative indices of the relative frequency of citations (RFC) and informant consensus factor (ICF) were calculated to evaluate the plant species that were utilized by the community.

**Results:**

A total of 28 medicinal plants from 20 different families and 6 ritual plants from 5 different families were documented. Asteraceae, Poaceae, and Lamiaceae (10.71%) family are the most mentioned medicinal plant species, followed by Myrtaceae and Euphorbiaceae (7.14%). The most widely used growth form were herbs (46.4%), while leaves (61.5%) were the most utilized plant part, and the preparation of a decoction (62.2%) was the most preferred method of processing and application. The medicinal plants were most commonly utilized for wound-healing, cough and colds, stomachache and kidney trouble, whereas ritual plants were largely used for healing, protection, and funeral ceremonies.

**Conclusion:**

This study marks the first report on the medicinal and ritual plants used by a group of indigenous communities in Sto. Niño, Brgy. Ambassador, Tublay, Benguet Province. The data collected show that plant species belonging to the Asteraceae, Poaceae, and Lamiaceae family were the most mentioned and should be further evaluated by pharmacological analysis to assess their wider use for medicinal treatment.

**Supplementary Information:**

The online version contains supplementary material available at 10.1186/s41182-024-00624-1.

## Introduction

The use of medicinal plants identified in folklore for use as traditional medicines has been an integral part of history and culture throughout the globe [1], and forms an essential part of traditional medicine in the Philippines [2, 3]. This knowledge of such plants has been passed down from generation to generation through oral tradition [4, 5]. Historically, IPs in the Philippines have been using medicinal plants to treat ailments ranging from common ones, such as headache, stomachache, cough, colds, toothache, and skin diseases, to more serious and fatal ones, such as urinary tract infection, chicken pox, and dysentery [6]. These plants are still commonly used because they are believed to be efficient, safe, cost-effective, and accessible to local people and those who are living in rural and remote areas [7]. Additionally, cultural practices often use plants for spiritual activities, serving as offerings or essential ritual items [8]. Ritual plants possess symbolic meanings and serve spiritual functions in a wide range of ethnicities, religions, and belief systems among IPs. Understanding these indigenous beliefs is critical for integrating local community practices for the conservation of biodiversity [9].

The IPs have developed innovative uses for their locally sourced natural resources due to the accessibility of these plants. Traditional medication, adopted and passed down through generations by local healers, remains the basis of much of the healthcare in developing countries [7]. However, there is a noticeable decline in traditional knowledge, largely driven by factors like the movement of IPs from rural to urban regions, industrial growth, disappearance of natural environments, and lifestyle changes [10]. To avoid losing ethnobotanical knowledge, it is crucial to record and preserve it before it becomes irreversibly lost [11], especially as there are still limited written records for many local communities.

Ethnobotanical studies will preserve such indigenous plant-based knowledge, and ultimately conserve global heritage [12]. This information by providing baseline information benefits humanity and the scientific community especially since new globally transmitted diseases are increasing with evidence of resistance of problematic diseases to the current armory of pharmaceutical remedies. There is a growing need for potential alternative and nature-based sources of safe, effective, affordable medicine. Recent ethnobotanical studies in Camarines Sur [13], Iloilo City [14], and Butuan City [15], Philippines, documented numerous medicinal plants used to treat various ailments, with fever being the most common. However, these documented plants require further study to provide the testable scientific basis for their claimed efficacy and evaluation of their use as sources of effective plant-based drugs [10, 16–18].

This current study is part of the larger study of Bio + Mine project, funded through the Global Centre on Biodiversity for Climate (https://www.gcbc.org.uk/project/biodiversity-positive-mining-for-the-net-zero-challenge-biomine-project/) focused on the abandoned legacy mine site of Sto. Niño. Involving a consortium of partner institutions from the United Kingdom (Natural History Museum, and Imperial College of London) Philippines (De La Salle University, and Mindanao State University-Iligan Institute of Technology), and Australia (University of New South Wales Sydney) The project aims to develop a site-specific system for rehabilitation of the legacy mine site that fully integrates the local community’s existing knowledge, beliefs, and practices into co-designed outcomes for the site.

In Benguet province and the Cordillera region in Luzon, Philippines, where the Sto. Niño site is located, traditional knowledge regarding the use of plants is still common among older generations but scarcer among the younger generation; thus, there is a significant risk that this ethnobotanical knowledge may be permanently lost unless it is promptly and properly documented [6]. Recognizing this issue, this study aims to document, identify, and classify the medicinal and ritual plants that are used by the indigenous communities of Sto. Niño, Brgy. Ambassador, Tublay, Benguet.

## Materials and methods

### Study area

**Fig. 1:**
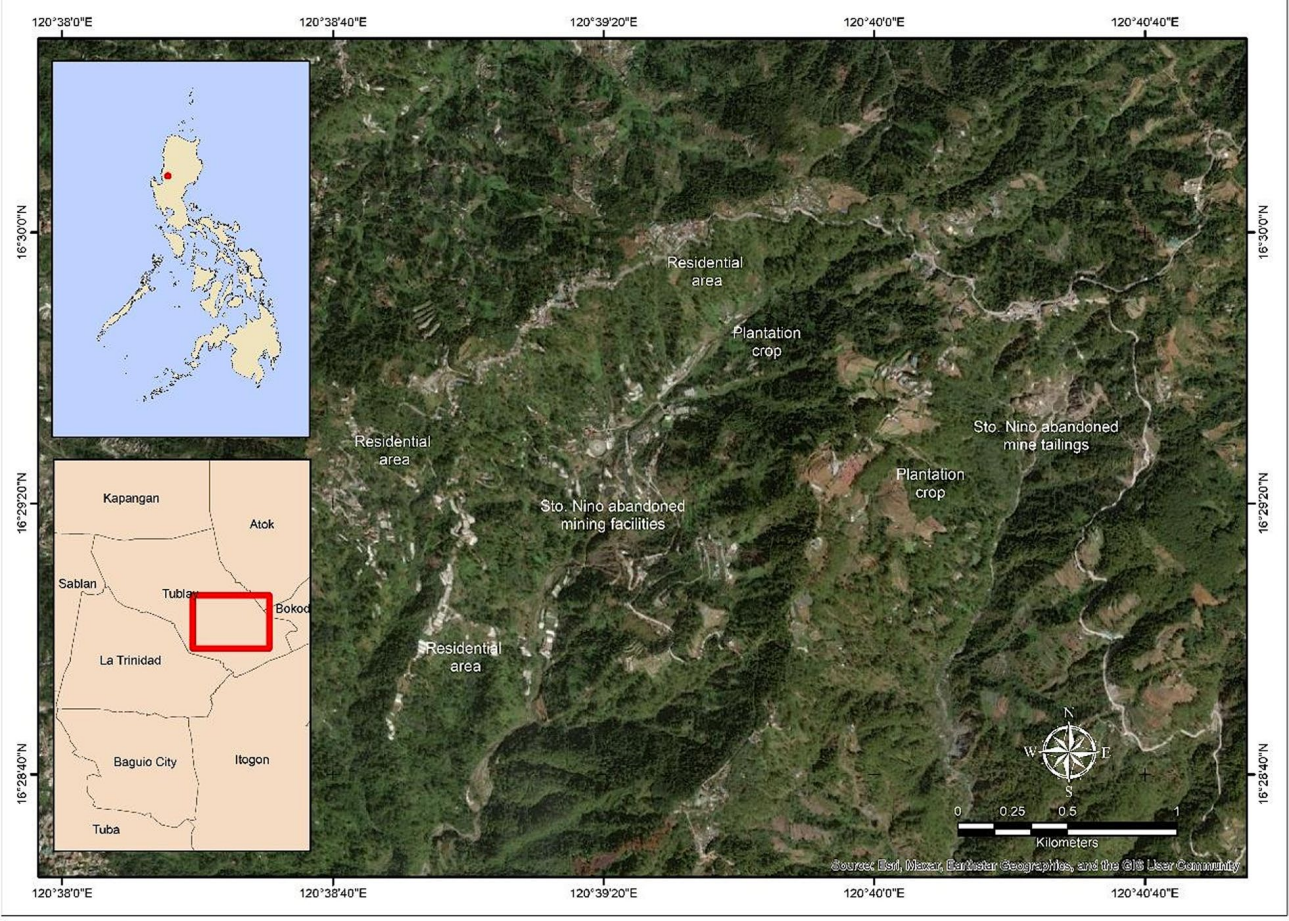
Map of Sto. Niño, Brgy. Ambassador, Municipality of Tublay, Benguet, Philippines. (Map is generated in ArcGIS 10.4; The Upper left corner is the Map of the Philippines with the study area highlighted; the lower left corner in a red-colored square is the Map of Benguet Province with the highlighted study area)

The study site is located in Sto. Niño, Brgy. Ambassador, Municipality of Tublay, Benguet, Philippines (16° 29′ 25'' N, 120° 39′ 20'' E; Fig. 1) [19]. The area is dominated by elevated and incised topography and variable vegetation cover with an estimated average elevation of around 5000 feet above sea level. The region has pronounced wet and dry seasons with mean temperatures ranging from the coldest point of around 6.5 °C to the warmest point of approximately 27.5 °C. Brgy. Ambassador is the largest barangay of the Municipality of Tublay, comprising 32% of the total land area of the municipality. This barangay is subdivided into 21 sitios; Aqueque, Amcaway, Babatan, Bayongabong, Belong, Central, Coroz, Ducot, King Solomon, Labey, Los-oc, Mamuyod, Nalseb, Pasanan, Patad-el, Pinanday, Sapu-an, Sub-ong, Tabeyo, Ulman, and Sto. Niño. The barangay contains many different cultures, beliefs and dialects and presently occupied by communities speaking Kankanaey (47.8%), Ibaloi (26%), Kalanguya (17.7%), and the smallest grouping is Ilocano (8%).

### Ethnobotanical data collection and identification

Data collection was conducted based on methods described by Alonzo et al. [19]. Prior to the collection of data, a community meeting was held to present the overall research design. This process is an integral part of the protocol set by Indigenous Knowledge Systems and Practices (IKSPs) and Customary Laws (CLs) Research and Documentation Guidelines of 2012 for working with Indigenous Peoples in the Philippines. This event was held by the research consortium together with the barangay officials, elders, and the residents of Sto. Niño, Brgy. Ambassador. All necessary permits were obtained, and ritual observations were conducted beforehand. The data were obtained through convenience and snowball sampling techniques, conducting semi-structured interviews and focus group discussions. A team member from the area was present to translate the conversations into the local dialect. A total of 100 locals were interviewed, with 12 individuals as the key interviewees.

The interview format was designed as informal conversations, encouraging respondents to speak spontaneously thus encouraging unfettered communication (Supplementary Fig. 4). Demographic information, such as age, gender, occupation/profession, and ethnobotanical knowledge (medicinal plants and their uses), was collected and anonymized or treated in accordance with data protection rules. Information, such as the medicinal plants used, the parts of the plant used, the mode of preparation, the mode of treatment, and the ailments treated, was recorded. Moreover, traditional knowledge and practices in ritual or spiritual activities were also documented. Plants were photographed for documentation and validation of morphological identification. Voucher specimens of uncommon plant species were also gathered for specimen processing and recording. Identification of the collected medicinal plants was made using online databases including Co’s Digital Flora of the Philippines (https://www.philippineplants.org/), Phytoimages (http://phytoimages.siu.edu/), Stuartxchange (https://www.stuartxchange.org/), and Plants of the World Online (http:// plantsoftheworldonline.org/). These identifications were then verified by a plant biologist from the Department of Biological Sciences, MSU-IIT, Philippines.

### Data analyses

Collected data were organized in the order of highest to lowest citation with its local name, scientific name, family, growth form, medicinal uses, part/s used, method of preparation, and mode and dosage of application. The results were analyzed by the following quantitative parameters.

### Relative frequency citation (RFC)

The RFC was used to quantify the frequency of use of certain species and was determined using the following formula:

$$\text{RFC}=\text{ FC}/\text{N}$$

where FC refers to the number of interviewees who mentioned a particular medicinal/ritual plant, and N represents the number of interviewees participating in the survey. RFC values vary from 0 to 1, and the higher the RFC value, the more important and valuable the plant is in the area. The RFC value was used to indicate the importance of each medicinal and ritual plant, and all surveyed plants were ranked in order of significance [20].

## Informant consensus factor (ICF)

The ICF was used to assess the homogeneity or degree of agreement of the interviewees’ knowledge about medicinal plants and calculated as follows:

$$\text{ICF}=(\text{Nur}-\text{Nt})/(\text{Nur}-1)$$

where Nur refers to the number of use reports or citations for each illness category, and Nt represents the number of species utilized in that specific category [21]. If the ICF value is 0, the use information is not exchanged among respondents, if the ICF is 1, the use information is exchanged among respondents [22].

## Results and discussion

### Demographic characteristics

A total of 100 respondents were interviewed which is shown in Table 1. 39% respondents were male and 61% were female participants. In terms of age, 42% of the respondents interviewed were between 20 and 40 years old, 43% between 41 and 60 years old, the highest frequency interviewed, 14% of the respondents interviewed were between 61 and 80 years old, and 2% were between 81 and 100 years old. The respondents can be subdivided into groups: farmers (57%), employees (24%), housewives (9%), community elders (7%), religious leaders (2%), and retired professionals (1%).

**Table 1:** Demographic profile of respondents from Sto. Niño, Brgy. Ambassador, Tublay, Benguet

Social group	Variables	No. of informants (n = 100)	Percentage (%)
Sex	*Female*	61	61
	*Male*	39	39
Age (years)	20–40	41	41
	41–60	43	43
	61–80	14	14
	81–100	2	2
Occupation	Farmer	57	57
	Employed	24	24
	Housewife	9	9
	Community elder	7	7
	Religious leader	2	2
	Retired professional	1	1

### Medicinal plants used by the local communities of Sto. Niño, Brgy. Ambassador

**Fig. 2:**
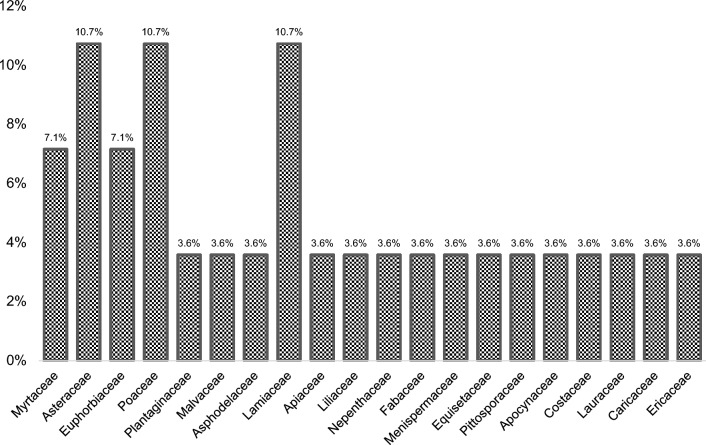
Family of the medicinal plants used by the IP communities of Sto. Niño, Brgy. Ambassador, Tublay, Benguet for medicinal application

A total of 28 medicinal plants belonging to 20 different families were documented in the ethnobotanical survey (Table 2). The results showed that Asteraceae, Poaceae, and Lamiaceae families (10.71%) were the most mentioned plant species (three plant species each family), in terms of frequency this is in line with the study of Hamidi et al. [23] who show that the families of Asteraceae, Lamiaceae, Poaceae, are the most commonly mentioned, plant families that are the most abundant in many ecosystems [24]. Also common are Myrtaceae and Euphorbiaceae (7.14%) (two plant species each family) (Fig. 2). The Poaceae family, with the most numbered plant species in this study, was also recorded in other ethnobotanical surveys conducted throughout the Philippines [25] and among IP communities in Panay Island, Morong Bataan [26], as well as in Iloilo and the adjacent province of Antique [27]. In the study of Al-Zubari et al., [28], it was reported that *Eleusine indica* (Poaceae: Poales) roots have depurative, diuretic, febrifuge, and laxative properties which is used to treat influenza, hypertension, oliguria, and urine retention. Our study found that *E. indica* is used by the IPs in Tublay, Benguet to treat high blood pressure or hypertension. The family Asteraceae has the most representative species among all plant families since it is one of the biggest and most diverse, with about 1,620 genera and 23,600 unique species [29]. This plant family is generally used by the IPs in Tublay, Benguet for treating wounds, and dysmenorrhea. This family of plants was found by previous works to possess anti-inflammatory, antibacterial, antioxidant, and other healing properties [30]. Several studies have been carried out on the bioactive substances found in this family identifying significant potential for pharmaceutical and medical uses [31]. The study by Muhammad et al. [32] outlined that the local communities use of this family of plants to treat a variety of human illnesses. The prevalence of the use of Asteraceae and Poaceae in Tublay suggests that these medicinal plants are growing and readily available in Tublay and this accessibility of these medicinal plants makes them popular for treating various ailments in IP communities. The continued reliance on these plant families indicates the persistence of traditional knowledge of medicinal plant use has been passed down through generations within these IPs community.

**Table 2:** Medicinal Plants used by the local communities of Sto. Niño, Brgy. Ambassador, Tublay, Benguet with their local or common name, scientific name, family, growth form, medicinal uses, and parts used, method of preparation, mode and dosage of application and its RFC value

Local or common name	Scientific name	Family	Growth form	Medicinal uses	Part/s used	Method of preparation	Mode and dosage of application (# times of time/day)	RFC
Bayabas	*Psidium guajava* L., NSM-3554	Myrtaceae	Tree	Wounds and skin disease	Leaves	Decoction	Drunk 2 times a day until healed	0.46
				LBM or stomachache	Shoot	Chewed	Chewed 3 times a day	
				Cough and cold	Young leaves	Decoction	Drunk once a day	
Oregano	*Origanum vulgare* L	Lamiaceae	Shrub	Cough	Leaves	Decoction	Drunk 3 times a day	0.25
				Urinary tract Infection (UTI) and migraine	Leaves	Decoction	Drunk 3 times a day	
Gumamela	*Hibiscus rosa-sinensis* L	Malvaceae	Shrub	Wound and inflammation	Flowers and Leaves	Crushed	Topical application until healed	0.21
Sangitan or Paragis	*Eleusine indica* (L.) Gaertn.,NSM-3658	Poaceae	Herb	High blood pressure	Whole plant	Decoction	Drunk when blood pressure is elevated	0.20
				Detoxification of the kidney	Whole plant	Decoction	Boiled for 10–15 min and drunk daily	
Aloe Vera	*Aloe barbadensis* Mill	Asphodelaceae	Herb	Detoxification of the digestive organ	Whole plant	Chewed	Chewed 3 times a day	0.17
				Ulcer, cough, hyperacidity, promote menstruation and wound-healing	Leaves	Direct eating	Scooped the aloe vera gel and eaten directly	
Bobongtit	*Ageratina riparia* (Regel) R.M.King & H.Rob.,NSM-3663	Asteraceae	Shrub	Wounds	Leaves	Crushed and Poultice	Topical application until the bleeding stops	0.15
Lanting	*Plantago major* L	Plantaginaceae	Shrub	Kidney trouble and fever	Whole plant	Decoction	Boiled for 15–20 min and drink once a day	0.15
Tagumbaw	*Jatropha curcas* L., NSM-3555	Euphorbiaceae	Shrub	Torn/detached nail and Rheumatoid arthritis	Bark	Heating	Topical application	0.12
				Anemia	Leaves	Decoction	Drunk 3 times a day	
Tawa-tawa	*Euphorbia hirta* L., NSM-3658	Euphorbiaceae	Herb	Dengue and Typhoid	Leaves and Roots	Decoction	Drunk daily	0.10
				Cough	Leaves	Steamed	Squeezed the steamed leaves and mix the 1 tbsp of extract with honey or lemon juice	
Lemon grass	*Cymbopogon citratus* DC	Poaceae	Herb	Cough	Whole plant	Decoction	Drunk 3 times a day	0.10
				Detoxification of the kidney	Leaves	Decoction	Drunk 3 times a day	
Takip kohol or Gotu kola	*Centella asiatica* L., NSM-3669	Apiaceae	Herb	Cough	Whole plant	Decoction	Drunk daily	0.08
				Kidney stone and UTI	Leaves and Stem	Decoction	Drunk daily	
Herbaca	*Artemisia vulgaris* L..NSM-3660	Asteraceae	Herb	Dysmenorrhea	Whole plant	Crushed or Decoction	Drunk 2–3 times a day pure extract (1 tbs)	0.07
Trumpet lily	*Lilium longiflorum* Thunb., NSM-3670	Liliaceae	Herb	Scabies	Leaves	Decoction	Drunk once a day use when taking a bath	0.07
Gaon or Cogon	*Imperata cylindrica* L., NSM-3664	Poaceae	Herb	Stomachache	Young Leaves	Crushed	Resin is rubbed	0.06
Makahiya	*Mimosa pudica* L	Fabaceae	Herb	UTI	Leaves	Decoction	Drunk daily	0.06
Makabukay	*Tinospora crispa* (L.) Hook.f. & Thomson., NSM-3628	Menispermaceae	Climber	Hypertension	Stem	Decoction	Drunk daily	0.06
Sunflower	*Tithonia diversifolia* (Hemsl.) A.Gray., NSM-3665	Asteraceae	Herb	Wound-healing	Leaves	Crushed	Once via topical application	0.05
Dara-dara	*Iresine diffusa*, NSM-3666	Lamiaceae	Shrub	Sore eyes	Leaves	Decoction	Face wash 3 times a day	0.05
Talubotob	*Equisetum ramosissimum* Desf., NSM-3668	Equisetaceae	Herb	Cough and UTI	Leaves	Decoction	Steam inhalation and drunk daily	0.05
Avocado	*Persea americana* Mill	*Lauraceae*	Tree	LBM	Leaves	Decoction	Drunk 3 times a day	0.02
Lagundi	*Vitex negundo* L	Lamiaceae	Herb	Cough	Leaves	Decoction	Drunk 3 times a day	0.02
Serpentina	*Rauvolfia serpentina* (L.) Benth. ex-Kurz., NSM-3661	Apocynaceae	Shrub	Cancer and heart disease	Leaves	Decoction	Drunk 1 glass a day	0.01
Papaya	*Carica papaya* L	Caricaceae	Tree	Dengue	Leaves	Decoction	Drunk 3 times a day	0.01
Eucalyptus	*Eucalyptus* sp.	Myrtaceae	Shrub	Cough and Colds	Whole plant	Decoction	Drunk 2 times a day and used for bath	0.01
Taugtug	*Gaultheria cumingiana* (Turcz.) Sleumer., NSM-3667	Ericaceae	Shrub	Wounds	Leaves	Crushed	Topical application	0.01

**Fig. 3:**
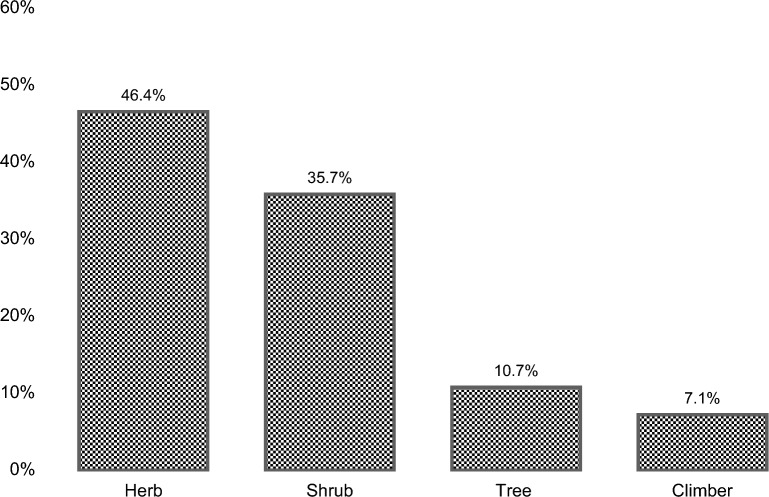
Growth form of plants (herb, shrub, tree, and climber) used by the IP communities of Sto. Niño, Brgy. Ambassador, Tublay, Benguet for medicinal application

**Fig. 4:**
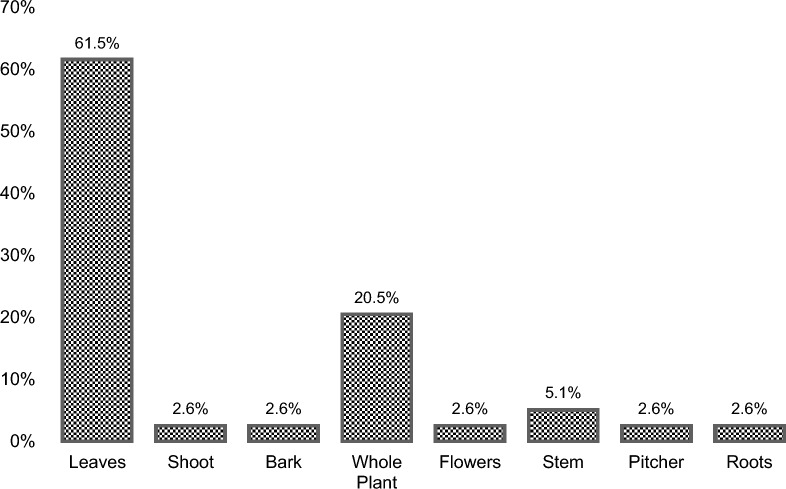
Plant parts (leaves, shoot, bark, whole plants, flowers, stem, pitcher, and roots) used by the IP communities of Sto. Niño, Brgy. Ambassador, Tublay, Benguet for medicinal application

In terms of type, the medicinal plants recorded in this study were mainly herbs (46.4%), shrubs (35.7%), trees (10.7%) and climbers (7.1%) (Fig. 3), a distribution consistent with the previous works of Caunca and Balinado [33]. The different plant parts that were used in medicinal practices include leaves, roots, stems, bark, whole plant parts, and flowers (Fig. 4). The most consumed plant part was found to be the leaves (61.5%) followed by the whole plant (20.5%), stem (5.1%), shoot (2.6%), bark (2.6%), flowers (2.6%), and pitcher (2.6%). Leaves are frequently used in ethnobotanical research [34] because they store large amounts of chemical compounds through photosynthesis, which concentrates active components [35]. According to Hamel et al. [36], the synthesis of many secondary metabolites occurs in leaves which would explain the relatively frequent use of plant leaves in comparison to other parts of plants. Moreover, using leaves is a sustainable practice in the long term and promotes better ecological balance compared to the harvesting of other parts like roots [37], and so utilizing leaves for medicinal purposes supports conservation management of plants, supporting sustainable harvesting practices while maintaining biodiversity and ecological balance.

**Fig. 5:**
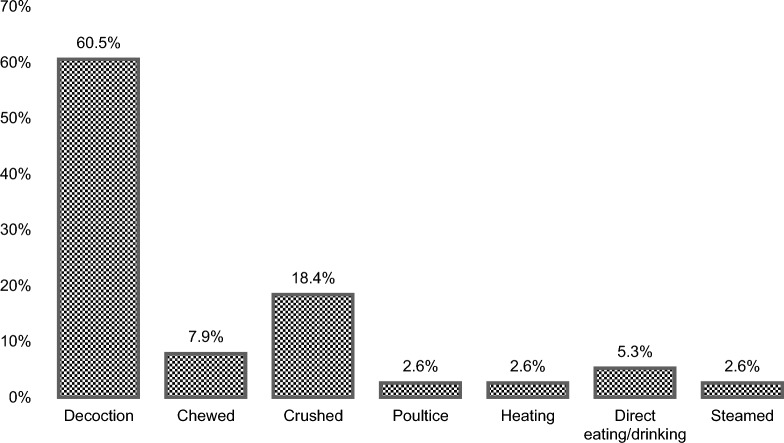
Mode of preparation (decoction, chewing, crushing, poultice, heating, direct eating/drinking, and steaming) of medicinal plants used by the IP communities of Sto. Niño, Brgy. Ambassador, Tublay, Benguet

Decoction, steaming, crushing, chewing, drinking and boiling were some of the modes of preparation documented in this survey (Fig. 5). Decoction (60.2%) was most often mentioned and the most common mode of application of the medicinal plants followed by crushing (18.4%), chewing (7.9%), direct eating/drinking (5.3%), poultice (2.6%), heating (2.6%), and steaming (2.6%). The findings are supported by previous publications, where decoction was suggested to be the preferred method of preparation by the IP groups in the Philippines [38]. The most popular method of administration, particularly for severe conditions, is drinking the decoction, as the active components in the decoction can enter the body more quickly, resulting in the strongest effect [39]. Decoction is straightforward, but its preparation is time-consuming, has to be freshly prepared for maximum effects, and occasionally yields an unpleasant taste to the medicine [40].

*Psidium guajava* had the highest RFC value (0.46) among the medicinal plants recorded. Chewing of the young leaves and shoots of this plant or drinking of plant extracts from decoction drinking is commonly practiced to treat various ailments, such as wounds and skin diseases, loose bowel movement (LBM) stomachache, cough and colds. In other parts of the world especially in subtropical regions, *P. guajava* is a popular food and as source of traditional medicine because of its known pharmacologic properties [41]. It has been documented for the treatment of a wide range of illnesses, including caries, hypertension, diabetes, diarrhea, fever, dysentery, and wounds [42]. More recent ethnomedicinal studies show that *P. guajava* is used in many parts of the world for the treatment of several diseases, e.g., as an anti-inflammatory, for diabetes, hypertension, caries, wounds, pain relief and reducing fever [43] as also used in this study. The bioactive components of *P. guajava* leaves include phenolic compounds, isoflavonoids, gallic acid, catechin, epicatechin, rutin, naringenin, and kaempferol. These compounds have been shown to have hepatoprotective, antioxidant, anti-inflammatory, anti-spasmodic, anti-cancer, antimicrobial, anti-hyperglycemic, and analgesic properties [44]. *P. guajava* leaves contain flavonoids that can lessen the severity of coughing [45]. Extracts from *P. guajava* leaves also have strong antibacterial properties that are shown to inhibit the growth of *Staphylococcus aureus* [46]. Methanolic extracts of *P. guajava*’s plant leaves and bark show strong antibacterial properties. These extracts have the ability to suppress *Salmonella* and *Bacillus* bacteria [47].

*Origanum vulgare* has the second highest RFC value of 0.25 that was used in treating urinary tract infections and migraine. Therapeutic benefits for *O. vulgare* expand back to the ancient Greek and Roman empires where applications of the leaves were used to cure skin ulcers, to ease aching muscles, and as an antibacterial. *O. vulgare* also has been utilized in traditional treatments for such diseases as asthma, cramps, diarrhea, and indigestion [48]. In the Philippines, *O. vulgare* is reported also for its medicinal properties. According to a study by Orillaneda & Acero [49], it is used by middle-aged residents of San Antonio, Tandag, Surigao City, to treat a variety of conditions, including malaria, liver problems, calculi linked to renal diseases, cough, chronic asthma, bronchitis, against helminths, colic, convulsions, and epilepsy. The Higaonon populations of Sitio Lomboyan, Barangay Guinabsan, Buenavista, Agusan del Norte were also documented in the study by Omac et al. [50] to have used oregano in treating various ailments. *Origanum p*lants exhibited numerous biological activities, including antimicrobial, anticancer, antidiabetic, antinociceptive, insecticidal, hepatoprotective, cytotoxic, and antilipase properties [51]. Flowers, leaves, and stems of *O. vulgare* were used to extract essential oils, which were found to contain β-caryophyllene as well as other components like 1,8-cineole, α-pinene, and γ-cadinene [52]. *O. vulgare* essential oils have demonstrated antibacterial action, mainly against uropathogens *E. coli* and *Enterococcus* that are responsible for urinary tract infections. Thymol and carvacrol are primarily responsible for the antibacterial activities of oregano, with a lesser degree of contribution from their precursor monoterpenes, p-cymene and γ-terpinene [53]. In addition to being antibacterial, antifungal, antiproliferative, and analgesic, thymol can also neutralize free radicals and suppress proinflammatory chemicals. Antibacterial, antifungal, antiviral, immunomodulatory, antiproliferative, antioxidant, and anti-inflammatory properties are also present in carvacrol [54].

Lastly, *Hibiscus rosa-sinensis* with an RFC value of 0.21 is used to treat wounds and inflammation, applied topically in the wound area until the wounds are healed using its crushed flowers and leaves. The medicinal properties of *H. rosa-sinensis* were also reported by Abad et al. [55]. According to these authors, decoction of *H. rosa-sinensis* roots was used in Cagayan, Philippines for treating cough while its flowers and leaves are boiled to medicate the swelling of wounds. Similarly, Madjos & Ramos [1] reported that the Higaonon tribe in Sitio Lomboyan, Buenavista, Agusan del Sur utilizes its flower to treat boils by crushing and pasting it on the affected area. The leaf extracts are high enough in essential nutrients required for optimal physiological performance and the maintenance of good health. According to the study of Nayak et al. [56], tannins are plant metabolites that aid in the healing of wounds. The antibacterial properties of the leaves' solvent and aqueous extracts, *H. Rosa sinensis* are ascribed to the existence of certain bioactive substances possessing antimicrobial characteristics. The presence of various antibacterial agents (flavonoids, tannins, alkaloids, and terpenoids) in flower extracts of *H. rosa-sinensis* was reported by Agarwal and Prakash [57]. Many biological processes and pharmacological qualities are associated with the bioactive compounds found in plants that can form as secondary metabolites [58].

This ethnomedicinal survey showed that the local communities of Sto. Niño, Brgy. Ambassador, Tublay, Benguet are still using some plant species that have medicinal properties to treat a range of ailments from wounds and skin disease to more serious diseases, such as hypertension, cancer and kidney disease.

### Informant consensus factor

The culturally significant medicinal plants were highlighted by calculation of an informant consensus factor (measures the level of homogeneity of responses for the plants to be used for each ailment category). Under 12 different disease categories, the study listed 28 diseases (Table 3). The listed ICF values ranged from (0.33) to (0.89) genitourinary diseases (kidney problem, urinary tract infection, and detoxification) had the highest ICF value (0.89) and blood diseases (anemia and menstrual problem) had the lowest ICF value (0.33). *Centella asiatica* is the most used medicinal plant species for treatment in genitourinary diseases. Heinrich et al. [22] state that to determine which species are more likely to contain intriguing bioactive components, a high ICF value is required. Choosing which species to keep in a setting where the number of therapeutic plant species is steadily declining requires a high informant consensus factor.

**Table 3:** Disease category and the ICF values of plants from Sto. Niño, Brgy. Ambassador, Tublay, Benguet

Illness category	Disease and ailment under each category	Number of plant species used	ICF
Respiratory diseases	Cough	9	0.83
Genitourinary diseases	Kidney problem, urinary tract infection, and detoxification	8	0.89
Digestive diseases	Stomachache, diarrhea, loose bowel movement, ulcer, hyperacidity, toothache, and detoxification	6	0.83
Injury	Cuts, Wounds, and inflammation	6	0.85
Cardiovascular diseases	Hypertension, high blood pressure, and heart disease	3	0.78
Blood diseases	Anemia and menstrual problem	3	0.33
General symptoms	Cold and fever	3	0.75
Infectious disease	Sore eyes, typhoid, and dengue	3	0.50
Skin diseases	Scabies and skin problem	2	0.67
Connective diseases	Rheumatoid arthritis	1	0
Metabolic disease	Diabetes	1	0
Nervous system disease	Migraine	1	0

### Ritual plants used by the local communities of Sto. Niño, Brgy. Ambassador

A total of six plant species were documented and identified by the participants that belong to five families that are used in ritual practices. Table 4 summarizes the plant information, plant part/s used and the ritual ceremonies in which these plants were used.

**Table 4:** Ritual plants used by the local communities of Sto. Niño, Brgy. Ambassador, Tublay, Benguet with local or common names, scientific names, family, growth form, plant parts used, and ritual ceremonies

Local or Common Name	Scientific Name	Family	Growth form	Plant part/s used	Ritual ceremonies
Dengaw	*Acorus calamus* L	Acoraceae	Herb	Roots or Leaves	Healing and protection
Tanapo	*Angiopteris evecta* (G.Forst.) Hoffm	Marattiaceae	Herb	Trunk	Healing and protection
Bellang	*Miscanthus* sp.	Poaceae	Herb	Stick	Protection
Luya	*Zingiber* sp.	Zingiberaceae	Herb	Fruit	Protection and success
Pinit	*Rubus* sp.	Rosaceae	Shrub	Leaves and thorns	Funeral
Salin	*Pennisetum* sp.	Poaceae	Herb	Leaves	Funeral

*Acorus calamus* can be used to relieve headaches and stomach pain when someone passes by a cemetery and to protect against evil spirits. Bracelets made from the dry roots or leaves of this plant are worn at all times, especially by babies and children. The person wearing the bracelet would pray when experiencing pain, or someone would pray over them. This plant is also documented in the study of Bersamin et al. [10], where it was recorded to have been used as an amulet to drive away evil spirits. *A. calamus* is an important aromatic medicinal plant and is used by the Ybanag Ethnic Minority in the Northern Cagayan Valley for wound-healing [59], utilized by the local people of Agusan del Sur for treating cold [60], and for stimulation of menstrual period and cycle by the Muslim Maranaos in Iligan City [61]. Previous studies have shown high antimicrobial properties of the plant against yeast and other fungi [62], which may explain why topical application of the plant extracts promote wound-healing [63].

*Angiopteris evecta* is used to relieve pain and ward off evil spirits. The trunk of the plant is cut and shaped like a human head to represent the person experiencing pain. The ritual ceremonies involve praying, dancing, and finally throwing a spear into the carved head. This plant was also documented for its medicinal uses, for instance, the Manobo Tribe in Agusan del Sur province used this to treat muscle pain [64]. Studies also showed that the ethanolic extract of the roots from this species has hypoglycemic properties [65]. The leaf extracts have antioxidant and antinociceptive properties [66–68].

*Miscanthus sinensis* is used to ward off evil spirits. The ritual involves placing a small stick on the back of the affected person. The Kankanaeys in Kibungan, Benguet use the stem of this plant to make hedges, and shoots of the plant are sometimes eaten raw [10]. It has also been used to promote wound-healing and skin whitening [69].

*Zingiber officinale* is also used for protection from evil spirits. The rhizome (underground stem) of this plant is sliced and placed inside a person’s pocket, much like storing a candy. This plant is also believed to bring success in life and prevent death. The fruit is thinly sliced, and the ritual process involve giving it to each person individually, similar to receiving a communion. This is also used by the local community in Kabayan, Benguet by placing the rhizome in an empty coffin prior to putting the dead body to disinfect it. Rhizomes are also used for medicinal purposes, mainly for colds and head-related illnesses [8]. This plant has been proven to have antiviral, anti-inflammatory, anticancer, and antioxidant properties [70].

*Rubus rosifolius* is used during funerals, and the ritual involved placing the leaves and thorns of this plant in the grave before placing inside the coffin to ward off bad spirits. This plant is used by the indigenous communities in Benguet and Ifugao for the treatment of stomachache, sore eyes, urinary tract infection, cough, wounds, and diarrhea [71]. The leaves of this plant are found to possess antioxidant properties [72], and aqueous leaf extracts are a potential anti-diabetic remedy, and moderate antibacterial activities [73].

Lastly, *Pennisetum* sp. is used after a funeral to bestow blessings upon those who attended. An elder performs the prayer, dips the leaves of these plants in water, and executes the ritual by sprinkling it on people.

## Conclusions and recommendations

Many plant species are still used regularly by the locals of Sto. Niño, Brgy Ambassador, Tublay, Benguet for medicinal and ritual uses. Plant species of the family Asteraceae, Poaceae, and Lamiaceae were the most common plant species deemed to have medicinal value. Most of the plant species belonging to this family have likely anti-inflammatory, antibacterial, antioxidant, and other healing properties which correlate with how the local population utilizes these plant species. The most commonly used growth forms of the medicinal plants used were herbs and shrubs and the most commonly consumed plant parts are the leaves with decoction, the usual method of preparation. Treatment generally involves drinking of a plant decoction until the patient is healed or until a significant reduction of symptoms is noted. The locals of Sto. Niño also use ritual plants which are commonly utilized for a mix of healing, protection, and funeral ceremonies. In conclusion, our ethnomedicinal survey documents varied plant species with medicinal value that are used and valued by the IPs of Sto. Niño, Brgy Ambassador, Tublay, Benguet, highly valued as treatments for specific ailments and diseases.

## Supplementary Information

Supplemntary file 1.

## Data Availability

All data generated during this study are included in this published article.

## Abbreviations

IP: Indigenous peoples
RFC: Relative frequency of citation
ICF: Informant consensus factor
LBM: Loose bowel movement
UTI: Urinary tract infection
*E. indica*: *Eleusine indica*
*P. guajava*: *Psidium guajava*
*O.** vulgare*: *Origanum vulgare*
*H.** rosa-sinensis*: *Hibiscus rosa-sinensis*
*A. calamus*: *Acorus calamus*
