# Supplementary material for: Ethnobotanical survey of medicinal and ritual plants utilized by the indigenous communities of Benguet province, Philippines
Source: Trop Med Health. 2024 Sep 10;52:59. doi: 10.1186/s41182-024-00624-1 (PMC11385124; doi:10.1186/s41182-024-00624-1)
Supplement: Supplementary file 1 — Supplemntary file 1. [file 41182_2024_624_MOESM1_ESM.docx]

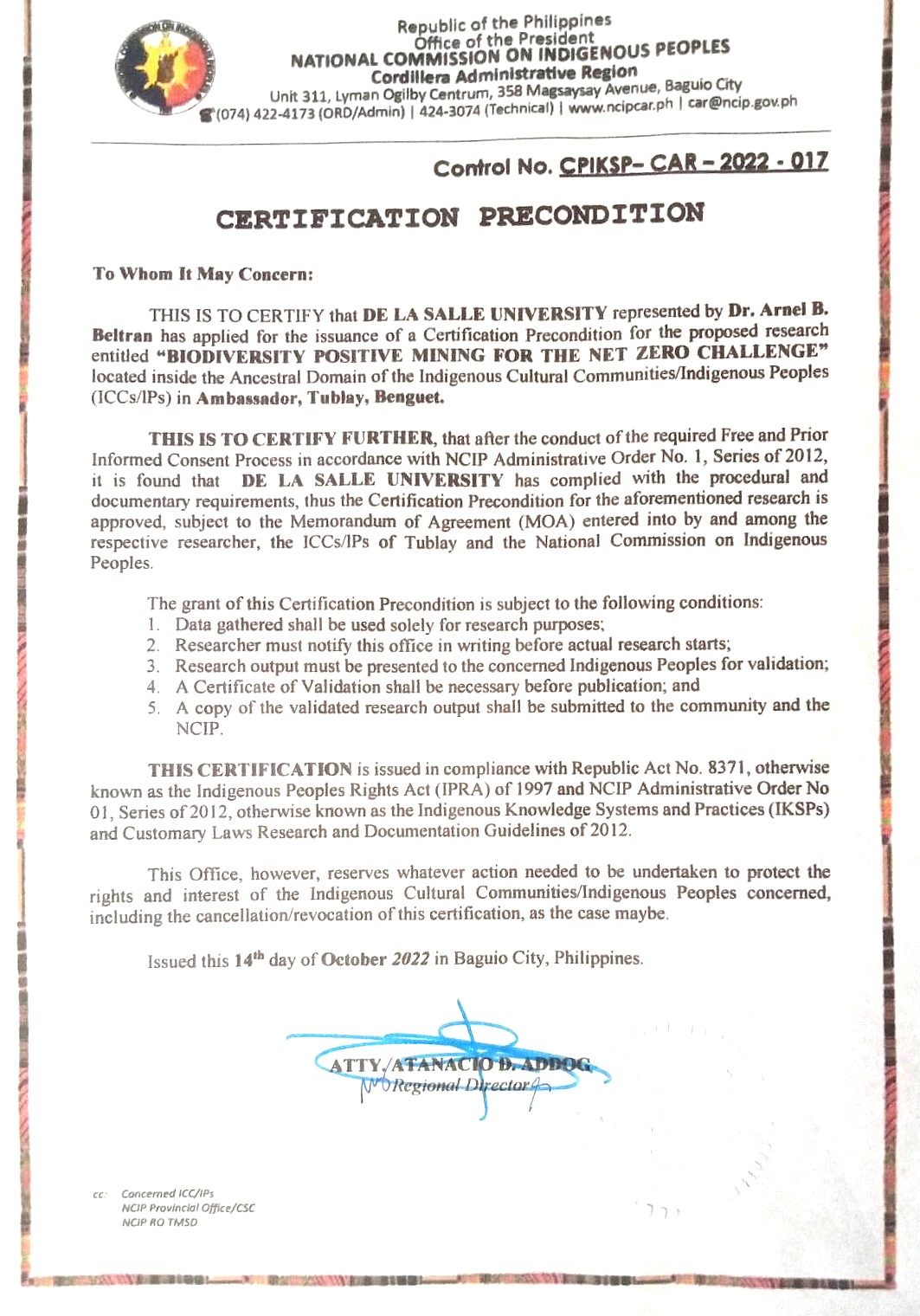


**Supplementary Figure 1.** Certificate of Precondition issued by the National Commission on Indigenous People (NCIP), Cordillera Administrative Region.


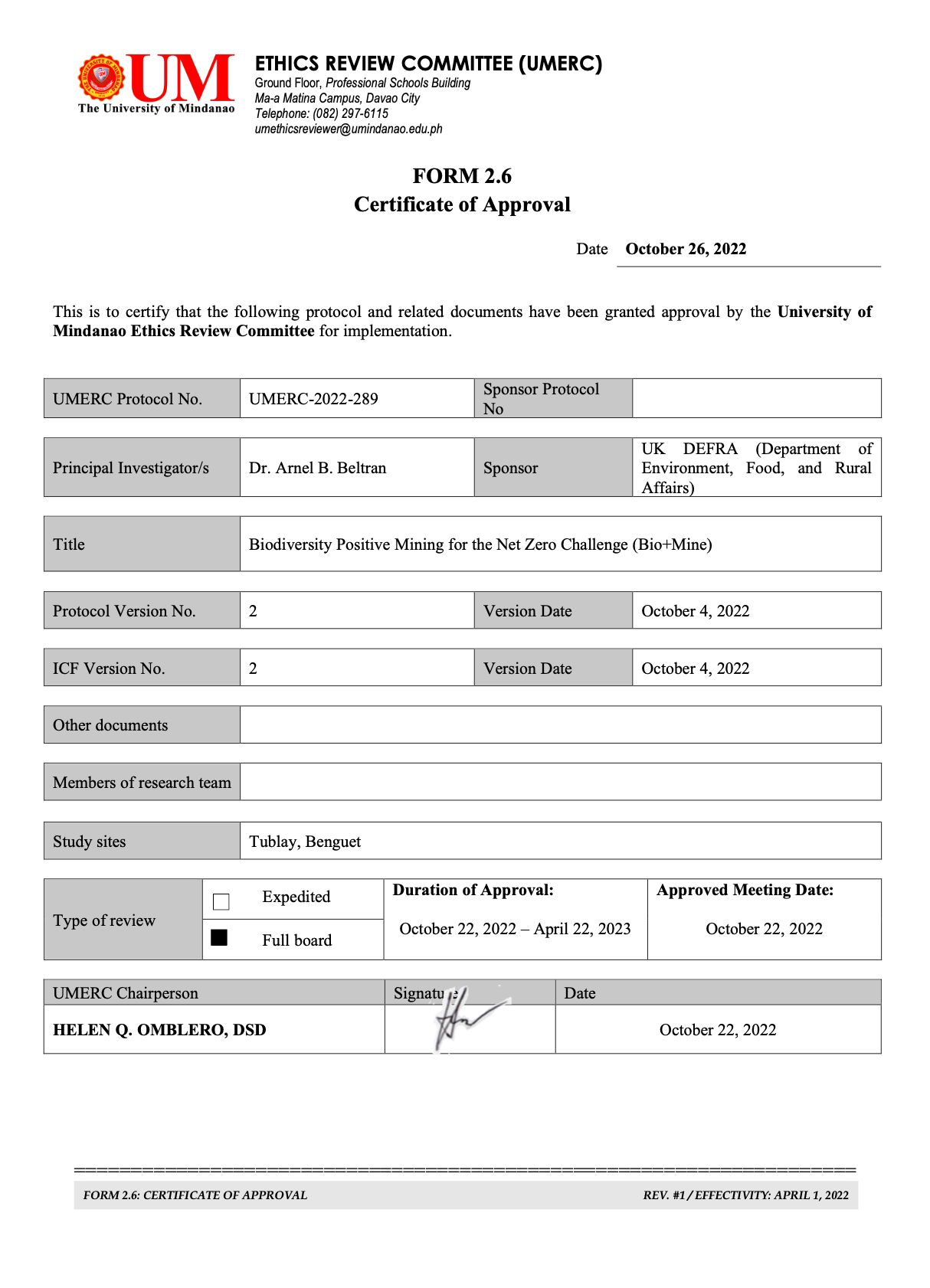


**Supplementary Figure 2.** Ethics approval granted by the University of Mindanao Ethics Review Committee, Davao City.


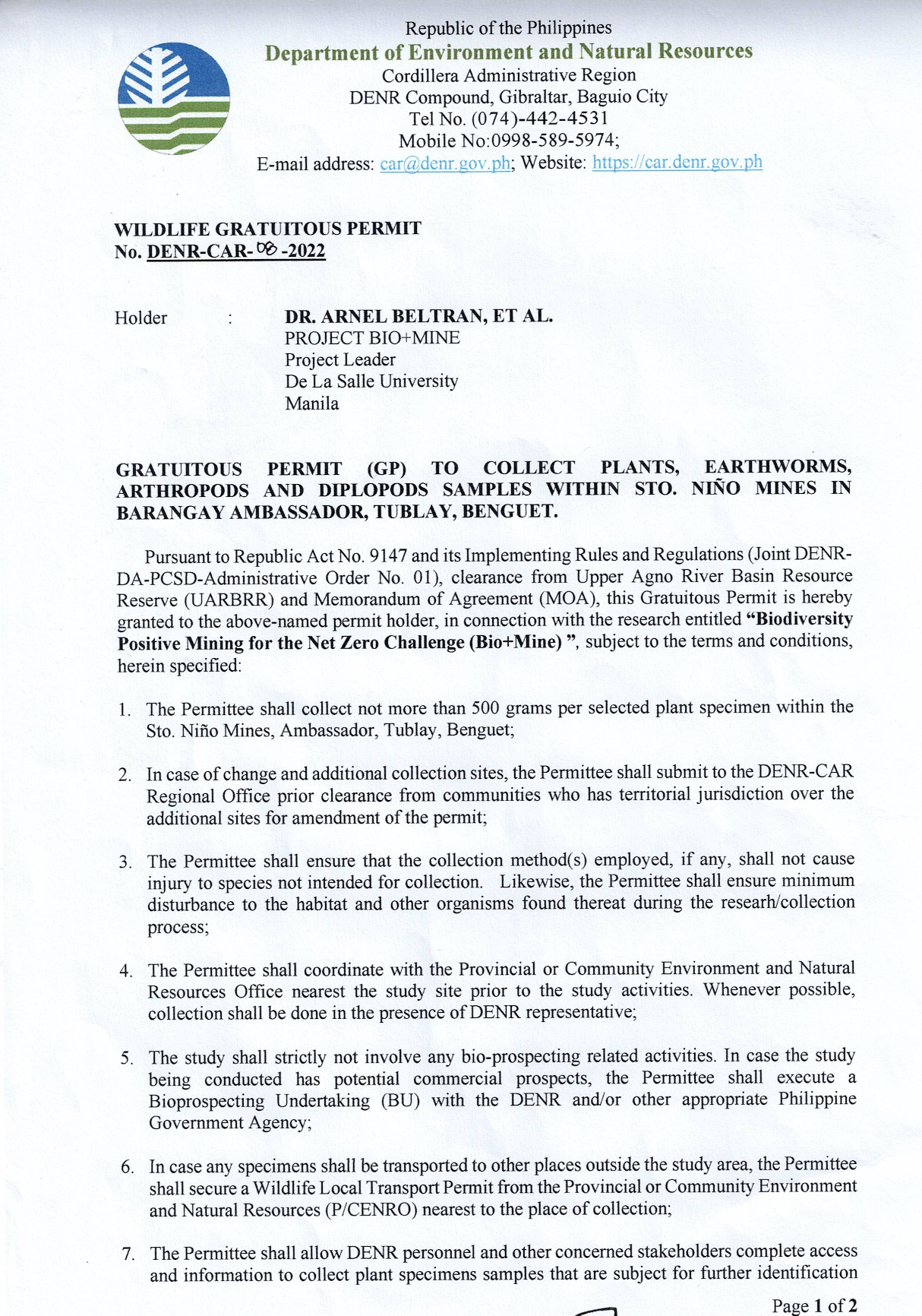


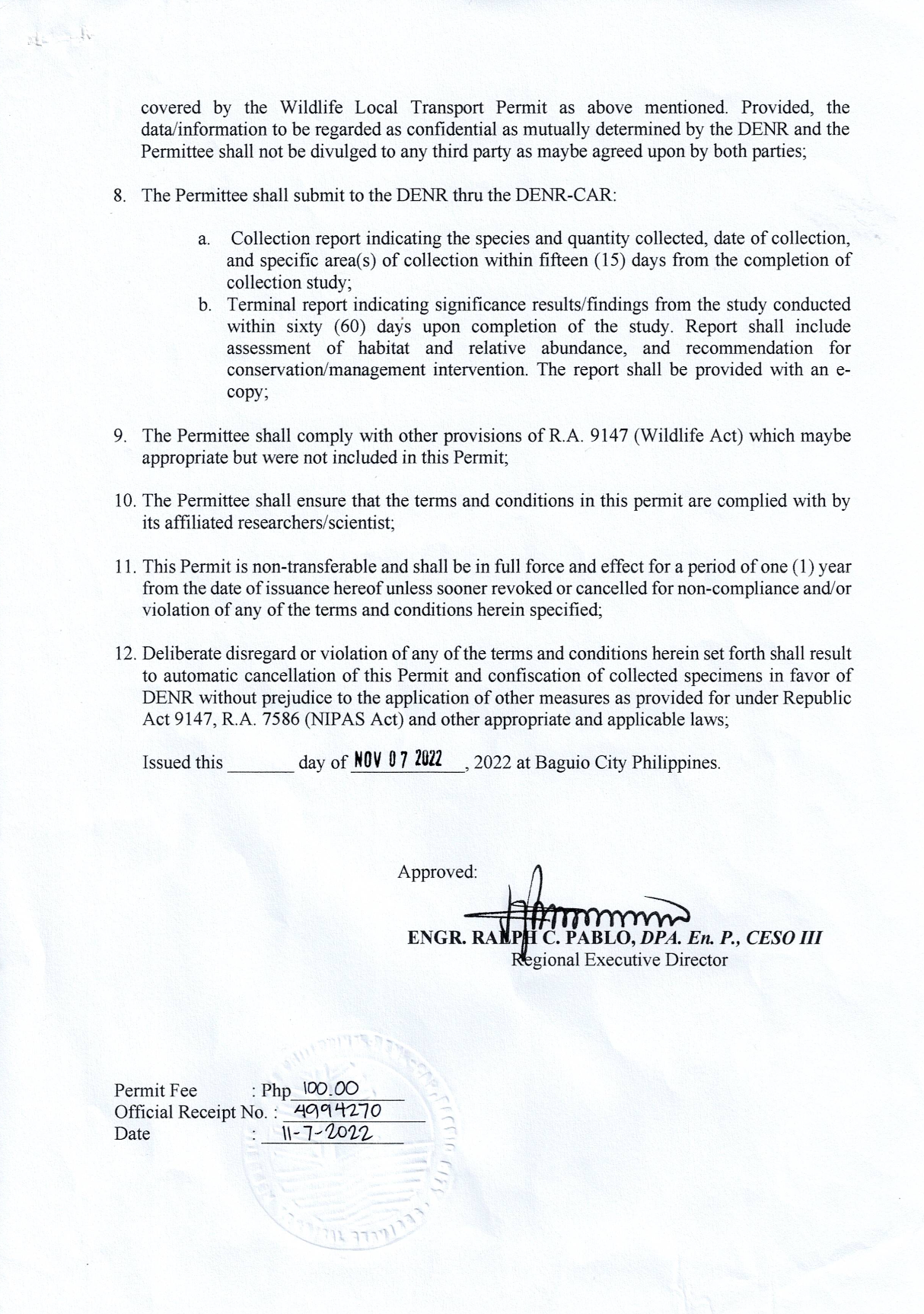


**Supplementary Figure 3.** Wildlife Gratuitous Permit issued by the Department of Environment and Natural Resources, Cordillera Administrative Region.


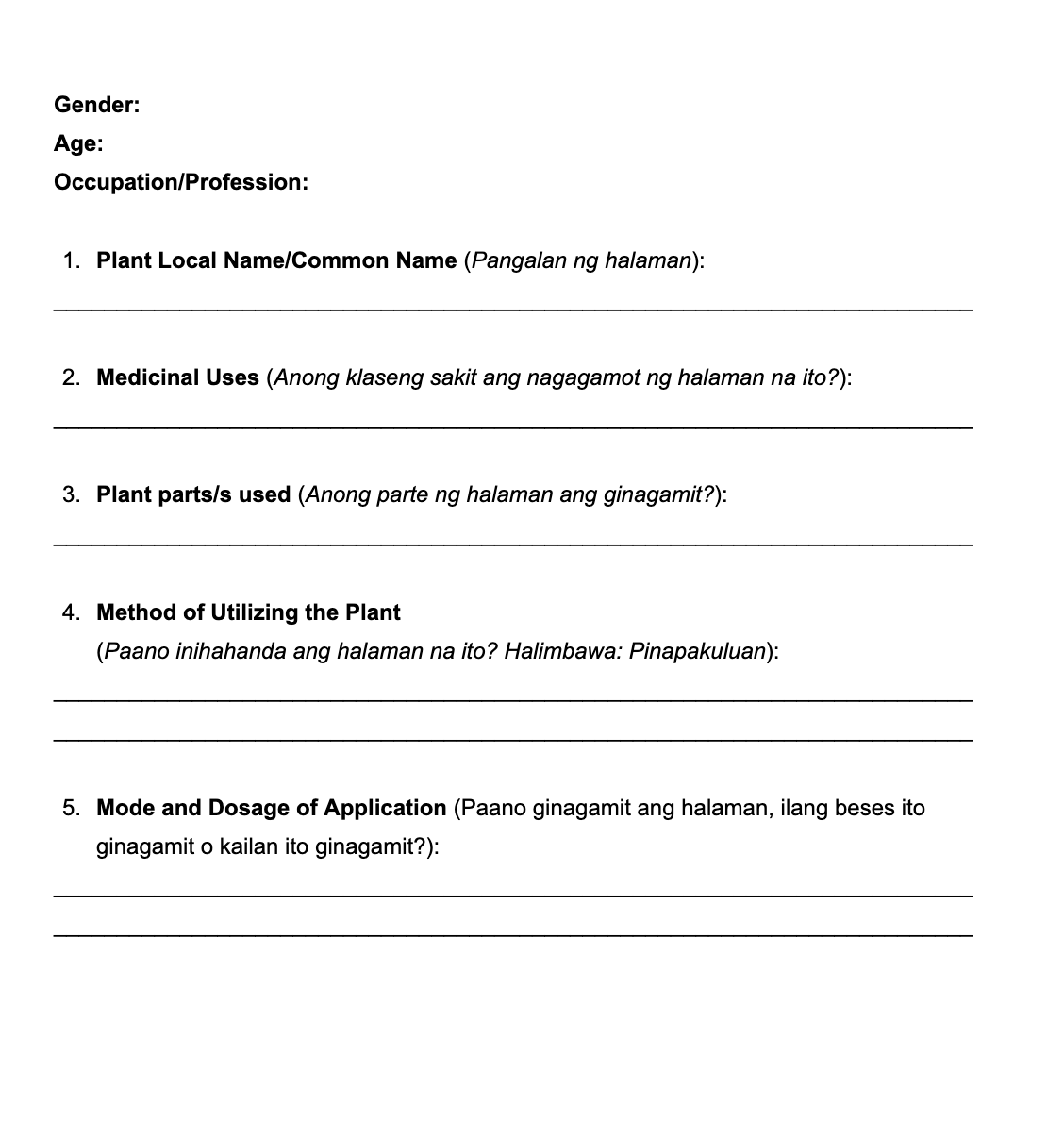


**Supplementary Figure 4.** Ethnobotanical survey questionnaire used during the collection of data.
